# [2-(Biphenyl-4-yl)-1,3-thia­zol-4-yl]methanol

**DOI:** 10.1107/S1600536812039062

**Published:** 2012-09-19

**Authors:** Manpreet Kaur, Jerry P. Jasinski, Amanda C. Keeley, H. S. Yathirajan

**Affiliations:** a Department of Studies in Chemistry, University of Mysore, Manasagangotri, Mysore 570 006, India; b Department of Chemistry, Keene State College, 229 Main Street, Keene, NH 03435-2001, USA

## Abstract

In the title compound, C_16_H_13_NOS, the central benzene ring makes dihedral angles of 3.25 (7) and 41.32 (8)°, respectively, with the thia­zole and phenyl rings. In the crystal, O—H⋯N hydrogen bonds link the mol­ecules into a chain along the *c* axis. A weak C—H⋯O inter­action further connects the chains into a layer parallel to the *ac* plane.

## Related literature

For pharmacological applications of thia­zole derivatives, see: Bishayee *et al.* (1997 ▶); Bhattacharya *et al.* (2005 ▶); Sharma *et al.* (2009 ▶). For the preparation of the title compound, see: Miyaura *et al.* (1979 ▶); Finholt *et al.* (1947 ▶). For related structures, see: Ghabbour, Chia *et al.* (2012 ▶); Ghabbour, Kadi *et al.* (2012 ▶); Hökelek *et al.* (2006 ▶); Yathirajan *et al.* (2006 ▶). For bond-length data, see: Allen *et al.* (1987 ▶).
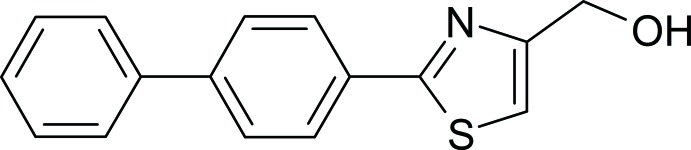


## Experimental

### 

#### Crystal data

C_16_H_13_NOS
*M* *_r_* = 267.33
Monoclinic, 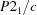

*a* = 6.05424 (18) Å
*b* = 29.3096 (9) Å
*c* = 7.2064 (2) Å
β = 92.668 (3)°
*V* = 1277.37 (7) Å^3^
*Z* = 4
Cu *K*α radiation
μ = 2.16 mm^−1^
*T* = 173 K
0.32 × 0.18 × 0.08 mm

#### Data collection

Oxford Diffraction Xcalibur (Eos, Gemini) diffractometer
Absorption correction: multi-scan (*CrysAlis RED*; Oxford Diffraction, 2010 ▶) *T*_min_ = 0.604, *T*_max_ = 0.841
7600 measured reflections
2501 independent reflections
2232 reflections with *I* > 2σ(*I*)
*R*_int_ = 0.050

#### Refinement

*R*[*F* ^2^ > 2σ(*F* ^2^)] = 0.041
*wR*(*F* ^2^) = 0.114
*S* = 1.05
2501 reflections
174 parameters
H-atom parameters constrained
Δρ_max_ = 0.30 e Å^−3^
Δρ_min_ = −0.27 e Å^−3^

### 

Data collection: *CrysAlis PRO* (Oxford Diffraction, 2010 ▶); cell refinement: *CrysAlis PRO*; data reduction: *CrysAlis RED* (Oxford Diffraction, 2010 ▶); program(s) used to solve structure: *SHELXS97* (Sheldrick, 2008 ▶); program(s) used to refine structure: *SHELXL97* (Sheldrick, 2008 ▶); molecular graphics: *SHELXTL* (Sheldrick, 2008 ▶); software used to prepare material for publication: *SHELXTL*.

## Supplementary Material

Crystal structure: contains datablock(s) global, I. DOI: 10.1107/S1600536812039062/is5193sup1.cif

Structure factors: contains datablock(s) I. DOI: 10.1107/S1600536812039062/is5193Isup2.hkl

Supplementary material file. DOI: 10.1107/S1600536812039062/is5193Isup3.cml

Additional supplementary materials:  crystallographic information; 3D view; checkCIF report

## Figures and Tables

**Table 1:** Hydrogen-bond geometry (Å, °)

*D*—H⋯*A*	*D*—H	H⋯*A*	*D*⋯*A*	*D*—H⋯*A*
O1—H1⋯N1^i^	0.82	2.06	2.8618 (19)	166
C3—H3⋯O1^ii^	0.93	2.42	3.101 (2)	131

Symmetry codes: (i) 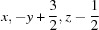; (ii) 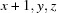.

**Fig. 1.**
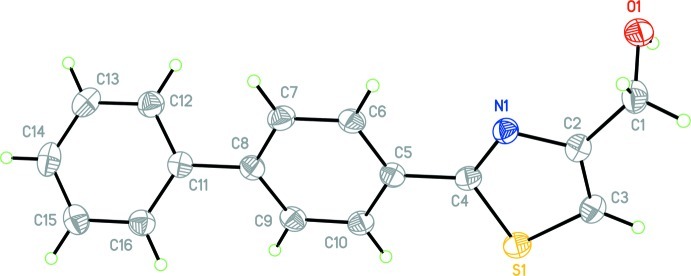
The molecular structure of the title compound with the atom numbering scheme. Displacement ellipsoids are drawn at the 50% probability level. H atoms are presented as small spheres of arbitrary radius.

**Fig. 2.**
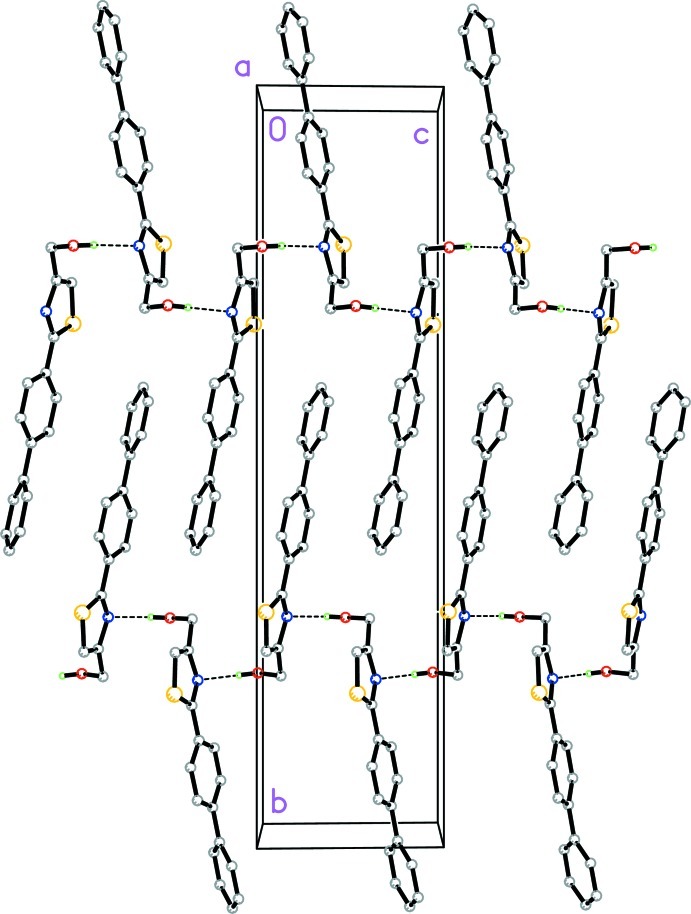
Packing diagram viewed along the a axis. O—H···N hydrogen bonds are shown by dashed lines forming infinite chains along the c axis. H atoms not involved in the hydrogen bonds have been omitted.

**Fig. 3.**
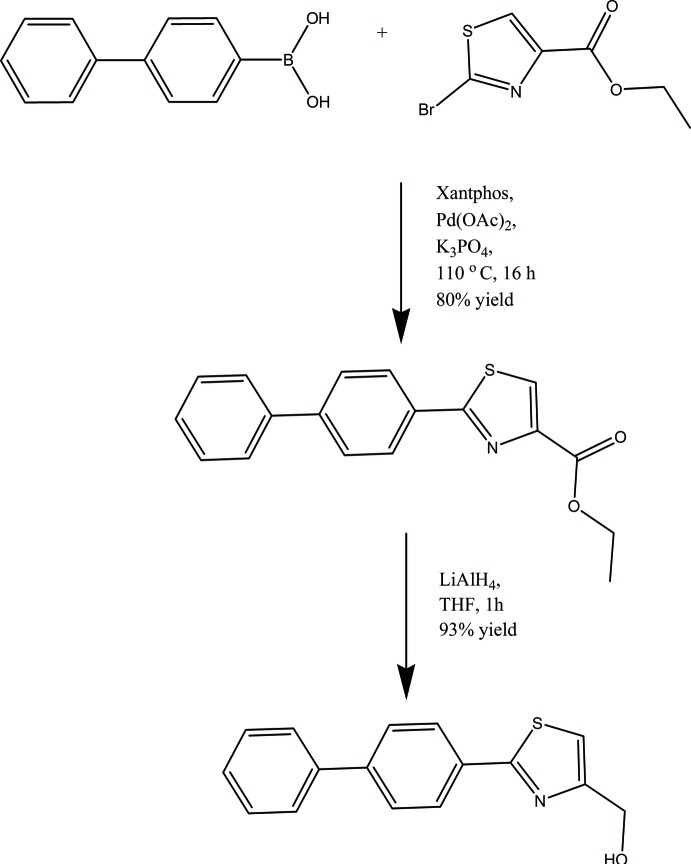
Experimental preparation summary of the title compound, C16H13NOS.

## supplementary crystallographic information

### Comment

Thiazole containing drugs have widespread use in a variety of medical conditions such as fungal and bacterial infections, gastric ulcers, cancer, etc (Bishayee *et al.*, 1997). Thiazole derivatives are involved frequently as the subject of drug design and synthesis efforts and they are reported to possess several activities like antibacterial, antifungal, anti-inflammatory (Sharma *et al.*, 2009), analgesic, antitubercular, central nervous system (CNS) stimmulant activity as well as anti-HIV activity (Bhattacharya *et al.*, 2005). Crystal structures of some thiazole derivatives, *viz.*, 3-(2-bromo-5-methoxyphenyl)-5-methyl-1-(4-phenyl-1,3-thiazol-2-yl) -1H-1,2,4-triazole (Yathirajan *et al.*, 2006), 2-amino-4-(4-methoxyphenyl)-1,3-thiazole (Hökelek *et al.*, 2006), 5-bromo-4-(3,4-dimethoxyphenyl)thiazol-2-amine (Ghabbour, Chia *et al.*, 2012) and N-[4-(4-bromophenyl)thiazol-2-yl]-4-(piperidin-1-yl)butanamide (Ghabbour, Kadi *et al.*, 2012) have been reported. In view of the importance of thiazoles, this paper reports the crystal structure of the title compound, (I), C_16_H_13_NOS.

In the title compound, (I), the dihedral angle between least-squares planes of the planar thiazole-phenyl unit (C2/C3/S1/C4/N1/C5–C10) and the outer phenyl ring (C11–C16) is 39.9 (5)° (Fig. 1). Bond lengths are in normal ranges (Allen *et al.*, 1987). O—H···N hydrogen bonds and weak C—H···O intermolecular interactions are observed which contribute to crystal packing forming a layer parallel to the *ac* plane (Fig. 2).

### Experimental

The title compound was prepared by the following procedure (Miyaura *et al.*, 1979; Finholt *et al.*, 1947). A mixture of 2-bromo-thiazole-4-carboxylic acid ethyl ester (1 g, 4.24 mmol), biphenyl boronic acid (1 g, 5.08 mmol), Xantphos (122.54 mg,0.212 mmol), tripotassium phosphate (2.6 g, 12.71 mmol) and palladium acetate (47.55 mg, 0.212 mmol) in THF as solvent was degassed for 15 mins and sealed. The sealed tube was heated at 383 K for 16 h under N_2_ atmosphere. After completion, the reaction mixture was diluted with ethyl acetate and filtered through a celite bed. It was then quenched with water and extracted with ethyl acetate. Organic layers were collected, dried over sodium sulphate and concentrated. Crude mass was purified through silica gel column chromatography (60:120 mesh) using 30% ethyl acetate in petroleum ether to afford 2-biphenyl-4-yl-thiazole-4-carboxylic acid ethyl ester (80% yield). Lithium aluminium hydride (122 mg, 3.23 mmol) was dissolved in minimum amount of THF in a RB flask and cooled to 0 °C under N_2_ atmosphere (Fig. 3). To this was added drop-wise a solution of 2-biphenyl-4-yl-thiazole-4-carboxylic acid ethyl ester (1 g, 3.23 mmol) in THF and stirred the reaction mixture for an hour. After completion of reaction, the reaction mixture was quenched with 10% sodium carbonate solution at 273 K and extracted with ethyl acetate. Organic layers were collected, dried over sodium sulphate and concentrated. Crude mass was purified through silica gel column chromatography (60:120 mesh) using 40% ethyl acetate in petroleum ether to afford the title compound (93% yield). X-ray quality crystals were obtained by slow evaporation of ethyl acetate solution (m.p.: 423–426 K).

### Refinement

All of the H atoms were placed in their calculated positions and then refined using the riding model with O—H = 0.82 Å and C—H = 0.93 Å (CH) or 0.97 Å (CH_2_). The *U*_iso_(H) values were set to 1.5*U*_eq_(O) and 1.2*U*_eq_(C).

### Crystal data

**Table:**

C_16_H_13_NOS	*F*(000) = 560
*M**_r_* = 267.33	*D*_x_ = 1.390 Mg m^−^^3^
Monoclinic, *P*2_1_/*c*	Cu *K*α radiation, λ = 1.54184 Å
Hall symbol: -P 2ybc	Cell parameters from 3467 reflections
*a* = 6.05424 (18) Å	θ = 3.0–72.5°
*b* = 29.3096 (9) Å	µ = 2.16 mm^−^^1^
*c* = 7.2064 (2) Å	*T* = 173 K
β = 92.668 (3)°	Chunk, colorless
*V* = 1277.37 (7) Å^3^	0.32 × 0.18 × 0.08 mm
*Z* = 4	

### Data collection

**Table:**

Oxford Diffraction Xcalibur (Eos, Gemini) diffractometer	2501 independent reflections
Radiation source: Enhance (Cu) X-ray Source	2232 reflections with *I* > 2σ(*I*)
Graphite monochromator	*R*_int_ = 0.050
Detector resolution: 16.0416 pixels mm^-1^	θ_max_ = 72.6°, θ_min_ = 3.0°
ω scans	*h* = −7→7
Absorption correction: multi-scan (*CrysAlis RED*; Oxford Diffraction, 2010)	*k* = −21→36
*T*_min_ = 0.604, *T*_max_ = 0.841	*l* = −8→8
7600 measured reflections	

### Refinement

**Table:**

Refinement on *F*^2^	Secondary atom site location: difference Fourier map
Least-squares matrix: full	Hydrogen site location: inferred from neighbouring sites
*R*[*F*^2^ > 2σ(*F*^2^)] = 0.041	H-atom parameters constrained
*wR*(*F*^2^) = 0.114	*w* = 1/[σ^2^(*F*_o_^2^) + (0.0662*P*)^2^ + 0.2333*P*] where *P* = (*F*_o_^2^ + 2*F*_c_^2^)/3
*S* = 1.05	(Δ/σ)_max_ = 0.001
2501 reflections	Δρ_max_ = 0.30 e Å^−^^3^
174 parameters	Δρ_min_ = −0.27 e Å^−^^3^
0 restraints	Extinction correction: *SHELXL97* (Sheldrick, 2008), Fc^*^=kFc[1+0.001xFc^2^λ^3^/sin(2θ)]^-1/4^
Primary atom site location: structure-invariant direct methods	Extinction coefficient: 0.0044 (7)

### Special details

**Table:**

Geometry. All esds (except the esd in the dihedral angle between two l.s. planes) are estimated using the full covariance matrix. The cell esds are taken into account individually in the estimation of esds in distances, angles and torsion angles; correlations between esds in cell parameters are only used when they are defined by crystal symmetry. An approximate (isotropic) treatment of cell esds is used for estimating esds involving l.s. planes.
Refinement. Refinement of *F*^2^ against ALL reflections. The weighted *R*-factor *wR* and goodness of fit *S* are based on *F*^2^, conventional *R*-factors *R* are based on *F*, with *F* set to zero for negative *F*^2^. The threshold expression of *F*^2^ > σ(*F*^2^) is used only for calculating *R*-factors(gt) *etc*. and is not relevant to the choice of reflections for refinement. *R*-factors based on *F*^2^ are statistically about twice as large as those based on *F*, and *R*- factors based on ALL data will be even larger.

### Fractional atomic coordinates and isotropic or equivalent isotropic displacement parameters (Å^2^)

**Table:**

	*x*	*y*	*z*	*U*_iso_*/*U*_eq_	
S1	0.63121 (7)	0.695240 (15)	0.03840 (6)	0.03231 (18)	
O1	−0.0110 (2)	0.78873 (4)	−0.02845 (18)	0.0324 (3)	
H1	0.0382	0.7907	−0.1323	0.049*	
N1	0.2361 (2)	0.70633 (4)	0.14310 (19)	0.0244 (3)	
C1	0.1660 (3)	0.78956 (6)	0.1071 (3)	0.0318 (4)	
H1A	0.1065	0.7906	0.2297	0.038*	
H1B	0.2524	0.8170	0.0911	0.038*	
C2	0.3140 (3)	0.74885 (5)	0.0952 (2)	0.0263 (4)	
C3	0.5237 (3)	0.74927 (6)	0.0367 (2)	0.0303 (4)	
H3	0.5986	0.7753	0.0008	0.036*	
C4	0.3844 (3)	0.67462 (6)	0.1183 (2)	0.0243 (3)	
C5	0.3507 (3)	0.62588 (5)	0.1571 (2)	0.0240 (3)	
C6	0.1540 (3)	0.61063 (5)	0.2309 (2)	0.0267 (4)	
H6	0.0440	0.6315	0.2567	0.032*	
C7	0.1218 (3)	0.56472 (6)	0.2657 (2)	0.0276 (4)	
H7	−0.0098	0.5552	0.3149	0.033*	
C8	0.2835 (3)	0.53247 (5)	0.2284 (2)	0.0244 (3)	
C9	0.4796 (3)	0.54796 (6)	0.1556 (2)	0.0269 (4)	
H9	0.5901	0.5271	0.1312	0.032*	
C10	0.5133 (3)	0.59363 (6)	0.1191 (2)	0.0268 (4)	
H10	0.6446	0.6030	0.0691	0.032*	
C11	0.2501 (3)	0.48303 (5)	0.2655 (2)	0.0249 (3)	
C12	0.0471 (3)	0.46207 (6)	0.2234 (2)	0.0298 (4)	
H12	−0.0698	0.4792	0.1725	0.036*	
C13	0.0174 (3)	0.41583 (6)	0.2567 (3)	0.0353 (4)	
H13	−0.1182	0.4022	0.2263	0.042*	
C14	0.1890 (3)	0.39001 (6)	0.3349 (3)	0.0351 (4)	
H14	0.1686	0.3591	0.3588	0.042*	
C15	0.3913 (3)	0.41044 (6)	0.3774 (2)	0.0328 (4)	
H15	0.5069	0.3932	0.4303	0.039*	
C16	0.4223 (3)	0.45643 (6)	0.3414 (2)	0.0281 (4)	
H16	0.5598	0.4697	0.3682	0.034*	

### Atomic displacement parameters (Å^2^)

**Table:**

	*U*^11^	*U*^22^	*U*^33^	*U*^12^	*U*^13^	*U*^23^
S1	0.0217 (3)	0.0331 (3)	0.0429 (3)	0.00099 (15)	0.00883 (18)	0.00499 (17)
O1	0.0255 (6)	0.0324 (7)	0.0403 (7)	0.0036 (5)	0.0110 (5)	0.0055 (5)
N1	0.0228 (7)	0.0239 (7)	0.0267 (7)	−0.0004 (5)	0.0034 (5)	0.0000 (5)
C1	0.0367 (10)	0.0228 (8)	0.0363 (9)	0.0001 (7)	0.0074 (7)	0.0000 (7)
C2	0.0275 (8)	0.0255 (8)	0.0259 (8)	−0.0023 (6)	0.0019 (6)	0.0002 (6)
C3	0.0286 (9)	0.0294 (9)	0.0330 (8)	−0.0040 (7)	0.0038 (7)	0.0040 (6)
C4	0.0212 (8)	0.0277 (8)	0.0243 (8)	−0.0007 (6)	0.0024 (6)	−0.0005 (6)
C5	0.0238 (8)	0.0249 (8)	0.0231 (7)	0.0011 (6)	0.0003 (6)	−0.0004 (6)
C6	0.0216 (8)	0.0264 (8)	0.0322 (8)	0.0045 (6)	0.0037 (6)	−0.0015 (6)
C7	0.0221 (8)	0.0295 (8)	0.0316 (8)	−0.0005 (6)	0.0042 (6)	−0.0011 (6)
C8	0.0253 (8)	0.0253 (8)	0.0223 (7)	0.0007 (6)	−0.0006 (6)	−0.0011 (6)
C9	0.0249 (8)	0.0280 (8)	0.0280 (8)	0.0059 (6)	0.0038 (6)	−0.0013 (6)
C10	0.0217 (8)	0.0300 (8)	0.0290 (8)	0.0013 (6)	0.0051 (6)	0.0007 (6)
C11	0.0272 (8)	0.0253 (8)	0.0223 (7)	0.0006 (6)	0.0035 (6)	−0.0023 (6)
C12	0.0280 (9)	0.0296 (8)	0.0320 (8)	0.0000 (7)	0.0025 (7)	−0.0005 (7)
C13	0.0330 (9)	0.0346 (9)	0.0390 (10)	−0.0080 (7)	0.0081 (8)	−0.0035 (7)
C14	0.0460 (11)	0.0249 (8)	0.0353 (9)	−0.0038 (7)	0.0125 (8)	0.0012 (7)
C15	0.0383 (10)	0.0286 (9)	0.0316 (9)	0.0045 (7)	0.0038 (7)	0.0022 (7)
C16	0.0278 (8)	0.0270 (8)	0.0295 (8)	0.0017 (6)	0.0009 (7)	−0.0021 (6)

### Geometric parameters (Å, º)

**Table:**

S1—C3	1.7120 (18)	C7—H7	0.9300
S1—C4	1.7350 (16)	C8—C9	1.396 (2)
O1—C1	1.416 (2)	C8—C11	1.489 (2)
O1—H1	0.8200	C9—C10	1.381 (2)
N1—C4	1.310 (2)	C9—H9	0.9300
N1—C2	1.382 (2)	C10—H10	0.9300
C1—C2	1.497 (2)	C11—C16	1.393 (2)
C1—H1A	0.9700	C11—C12	1.395 (2)
C1—H1B	0.9700	C12—C13	1.390 (3)
C2—C3	1.356 (2)	C12—H12	0.9300
C3—H3	0.9300	C13—C14	1.384 (3)
C4—C5	1.472 (2)	C13—H13	0.9300
C5—C6	1.400 (2)	C14—C15	1.385 (3)
C5—C10	1.400 (2)	C14—H14	0.9300
C6—C7	1.384 (2)	C15—C16	1.387 (2)
C6—H6	0.9300	C15—H15	0.9300
C7—C8	1.396 (2)	C16—H16	0.9300
			
C3—S1—C4	89.51 (8)	C9—C8—C7	117.93 (15)
C1—O1—H1	109.5	C9—C8—C11	120.60 (15)
C4—N1—C2	111.18 (14)	C7—C8—C11	121.47 (15)
O1—C1—C2	112.47 (14)	C10—C9—C8	121.50 (15)
O1—C1—H1A	109.1	C10—C9—H9	119.3
C2—C1—H1A	109.1	C8—C9—H9	119.3
O1—C1—H1B	109.1	C9—C10—C5	120.37 (16)
C2—C1—H1B	109.1	C9—C10—H10	119.8
H1A—C1—H1B	107.8	C5—C10—H10	119.8
C3—C2—N1	114.92 (15)	C16—C11—C12	118.34 (15)
C3—C2—C1	125.59 (15)	C16—C11—C8	120.65 (15)
N1—C2—C1	119.48 (15)	C12—C11—C8	121.01 (15)
C2—C3—S1	110.52 (13)	C13—C12—C11	120.78 (16)
C2—C3—H3	124.7	C13—C12—H12	119.6
S1—C3—H3	124.7	C11—C12—H12	119.6
N1—C4—C5	124.12 (15)	C14—C13—C12	120.21 (17)
N1—C4—S1	113.86 (12)	C14—C13—H13	119.9
C5—C4—S1	122.01 (12)	C12—C13—H13	119.9
C6—C5—C10	118.46 (15)	C13—C14—C15	119.53 (16)
C6—C5—C4	120.64 (14)	C13—C14—H14	120.2
C10—C5—C4	120.89 (15)	C15—C14—H14	120.2
C7—C6—C5	120.57 (15)	C14—C15—C16	120.35 (17)
C7—C6—H6	119.7	C14—C15—H15	119.8
C5—C6—H6	119.7	C16—C15—H15	119.8
C6—C7—C8	121.15 (16)	C15—C16—C11	120.78 (16)
C6—C7—H7	119.4	C15—C16—H16	119.6
C8—C7—H7	119.4	C11—C16—H16	119.6
			
C4—N1—C2—C3	−1.0 (2)	C6—C7—C8—C11	179.99 (15)
C4—N1—C2—C1	177.81 (14)	C7—C8—C9—C10	−0.8 (2)
O1—C1—C2—C3	109.65 (19)	C11—C8—C9—C10	179.58 (14)
O1—C1—C2—N1	−69.1 (2)	C8—C9—C10—C5	0.9 (2)
N1—C2—C3—S1	0.46 (19)	C6—C5—C10—C9	−0.6 (2)
C1—C2—C3—S1	−178.31 (14)	C4—C5—C10—C9	−179.82 (14)
C4—S1—C3—C2	0.15 (13)	C9—C8—C11—C16	40.4 (2)
C2—N1—C4—C5	179.93 (14)	C7—C8—C11—C16	−139.27 (17)
C2—N1—C4—S1	1.14 (17)	C9—C8—C11—C12	−138.87 (17)
C3—S1—C4—N1	−0.76 (13)	C7—C8—C11—C12	41.5 (2)
C3—S1—C4—C5	−179.58 (14)	C16—C11—C12—C13	0.1 (2)
N1—C4—C5—C6	−2.2 (2)	C8—C11—C12—C13	179.39 (15)
S1—C4—C5—C6	176.53 (12)	C11—C12—C13—C14	0.9 (3)
N1—C4—C5—C10	177.08 (15)	C12—C13—C14—C15	−0.9 (3)
S1—C4—C5—C10	−4.2 (2)	C13—C14—C15—C16	−0.2 (3)
C10—C5—C6—C7	0.1 (2)	C14—C15—C16—C11	1.3 (3)
C4—C5—C6—C7	179.41 (15)	C12—C11—C16—C15	−1.2 (2)
C5—C6—C7—C8	0.0 (3)	C8—C11—C16—C15	179.52 (15)
C6—C7—C8—C9	0.3 (2)		

### Hydrogen-bond geometry (Å, º)

**Table:**

*D*—H···*A*	*D*—H	H···*A*	*D*···*A*	*D*—H···*A*
O1—H1···N1^i^	0.82	2.06	2.8618 (19)	166
C3—H3···O1^ii^	0.93	2.42	3.101 (2)	131

Symmetry codes: (i) *x*, −*y*+3/2, *z*−1/2; (ii) *x*+1, *y*, *z*.
